# Optic Neuritis: The Influence of Gene Polymorphisms and Serum Levels of *STAT4* (rs10181656, rs7574865, rs7601754, rs10168266)

**DOI:** 10.3390/jcm13010010

**Published:** 2023-12-19

**Authors:** Greta Gedvilaite, Monika Duseikaitė, Gabrielė Dubinskaite, Loresa Kriauciuniene, Reda Zemaitiene, Rasa Liutkevicienė

**Affiliations:** 1 Laboratory of Ophthalmology, Institute of Neuroscience, Lithuanian University of Health Sciences, LT-50161 Kaunas, Lithuania; monika.duseikaite@lsmuni.lt (M.D.); loresa.kriauciuniene@lsmuni.lt (L.K.); rasa.liutkeviciene@lsmuni.lt (R.L.); 2 Medical Faculty, Lithuanian University of Health Sciences, LT-50161 Kaunas, Lithuania; gabriele.dubinskaite@stud.lsmuni.lt; 3 Department of Ophthalmology, Lithuanian University of Health Sciences, LT-50161 Kaunas, Lithuania; reda.zemaitiene@lsmuni.lt

**Keywords:** optic nerve, optic neuritis, *STAT4*, rs10181656, rs7574865, rs7601754, rs10168266, STAT4 ELISA

## Abstract

The aim of the study was to evaluate the associations of *STAT4* (rs10181656, rs7574865, rs7601754, rs10168266) gene polymorphisms and STAT4 serum level in patients with optic neuritis. Eighty-one subjects with optic neuritis (ON) and 158 healthy subjects participated in the study. Genotyping was performed using real-time polymerase chain reaction to obtain data. STAT4 serum level was determined using the ELISA method. Statistical analysis revealed that *STAT4* rs7574865 allele G was statistically significantly more frequent in patients with ON and multiple sclerosis (MS) than in the control group (84.38% vs. 65.93%, *p* = 0.003). *STAT4* rs10168266 allele C was statistically significantly more frequent in the ON group with MS than in the control group (89.06% vs. 71.75%, *p* = 0.003). The haplotypes G-G-A-C and C-T-A-T of *STAT4* (rs10181656, rs7574865, rs7601754, rs10168266) were associated with an 11.5- and 19.5-fold increased odds of ON occurrence (*p* = 0.003; *p* = 0.008, respectively). In optic neuritis without MS occurrence, *STAT4* (rs10181656, rs7574865, rs7601754, rs10168266) haplotypes G-G-A-C and C-T-A-T were found to be associated with 32.6- and 9-fold increased odds of ON without MS (*p* = 0.002, *p* = 0.016, respectively). The current findings may indicate a risk role of *STAT4* (rs10181656, rs7574865, rs7601754, rs10168266) G-G-A-C and C-T-A-T haplotypes in the occurrence of optic neuritis.

## 1. Introduction

The optic nerve, also known as cranial nerve II, is a structure of ganglion cell fibers that connects the brain with the retina and is responsible for transmission of visual information. The optic nerve, which is enveloped by three meninges (hard, soft, and arachnoid), is anatomically divided into four parts: the crystalline lens (*pars intraocularis*), the orbit (*pars intraorbitalis*), the part located in the cranial box (*pars intracranialis*), and the part of the nerve located in the bony canal (*pars intracanalicularis*) [1,2]. Due to its structure and location in the cranial cavity, the optic nerve is one of the most sensitive structures in the body and is most frequently damaged by various diseases, conditions, or injuries [3,4]. Changes in the optic nerve can also be a local sign of systemic diseases, such as idiopathic intracranial hypertension (IIH). In other cases, a para-physiological finding (optic nerve drusen) can simulate a serious pathology and mislead the clinician. For this reason, ultrasound examination of the optic nerve is of fundamental importance [5,6].

Under the influence of various environmental and genetic factors, visual impairment manifests itself in specific symptoms that may indicate possible damage to the aforementioned structure. One of the most common diseases of the optic nerve is optic neuritis (ON). This pathology usually occurs in people aged 18–45 years. The prevalence of optic neuritis is 1–5 cases per 100,000 population [3]. The best-studied and best-known causes of the disease are multiple sclerosis (MS), ischemia, infectious and autoimmune processes, so that ON can still be divided into typical and atypical forms [7,8].

Although the causes mentioned above are undoubtedly associated with optic nerve inflammation, the importance of genetic factors remains unclear. The influence of hereditary or congenital factors on the manifestation of certain diseases is increasingly recognized.

Members of the Signal Transducer and Activator of Transcription (STAT) proteins are responsible for physiological cellular processes in the body, such as proliferation, differentiation, apoptosis, angiogenesis, and the regulation of the immune system [9,10]. However, as with any other healthy part of the body system, genetic damage occurs, leading to inappropriate expression of STAT proteins. All this can be associated with pathological processes such as malignant cell transformation and metastasis [10]. The fourth member of the STAT family, STAT4, which is localized in the cytoplasm, can be phosphorylated by membrane-bound receptors, dimerized, and translocated to the nucleus, where it differentially regulates gene expression. This transcription factor transmits interleukin (IL)-12, (IL)-23, and type I interferon cytokine signals in T cells and monocytes. It leads to the differentiation of T helper type 1 and T helper type 17, the activation of monocytes, and the production of interferon, so the factor may be involved in many autoimmune diseases [11]. *STAT4* single nucleotide polymorphisms (SNPs) are known to be associated with an increased risk of autoimmune diseases, such as systemic lupus erythematosus (SLE), primary Sjogren’s syndrome (pSS), rheumatoid arthritis (RA), or thyroid disease [12], so an association with other less well-studied diseases is possible. Therefore, the aim of this study is to determine the associations of *STAT4* (rs10181656, rs7574865, rs7601754, rs10168266) with the manifestation of optic neuritis.

### 1.1. Clinic

The initial clinical manifestations are loss of vision in one eye, pain that worsens with eye movement, and impaired color perception [3]. Patients describe seeing as if through a fog, and initial changes can occur within a few hours to days. The earliest onset of dyschromatopsia (impaired color perception) affects a broad spectrum of colors [8]. As visual acuity can vary from minimal to complete vision loss, patients may complain of “flashes” of light during eye movements, known as photocopies [13,14]. When the depth perception of moving objects is altered, the disorder is referred to as Pulfrich phenomenon [15]. During the visual recovery phase, there may be a brief deterioration in vision after a hot shower or exercise (when body temperature rises)—this is known as the Unthoff symptom [16]. Central and peripheral ocular changes may occur during optic neuritis and are characteristic of 97% of patients. Central disturbances occur more frequently but recover more slowly. Almost all patients with bilateral ON have an afferent pupillary defect [14].

Based on the site of involvement, ON can be categorized as follows:

1. Retrobulbar neuritis with normal appearance of the optic disc;
2. Papillitis with a swollen optic disc;
3. Perineuritis, which affects the optic nerve sheath, while the optic disc may or may not be swollen;
4. Neuroretinitis with optic nerve oedema and macular exudates [17]. Retrobulbar neuritis and papillitis are mainly associated with MS, while perineuritis and neuroretinitis are more commonly associated with infectious or inflammatory pathologies [18].

### 1.2. STAT

Signal transducers and activators of transcription (STATs) are a family of proteins responsible for the essential and multifunctional regulation of physiological cellular processes, including proliferation, differentiation, apoptosis, angiogenesis, and immune regulation. As the name implies, these factors can transmit signals from the cell membrane to the cell nucleus and thereby activate gene transcription [9]. In their inactive form, STAT proteins are located in the cytoplasm. They become active through phosphorylation of tyrosine and serine amino acids. Dimers are already formed in the active state. Then they migrate to the nucleus, bind to DNA and regulate gene transcription [11,13,19]. Seven members of the STAT family have been identified in the human genome: STAT1, STAT2, STAT3, STAT4, STAT5A, STAT5B, and STAT6 [20]. Each STAT protein has its own function, and STAT3 and STAT5 are considered oncogenes [21].

### 1.3. STAT4 Gene

Several *STAT4* single nucleotide polymorphisms (SNPs) have previously been associated with an increased risk of autoimmune diseases such as systemic lupus erythemosus (SLE), primary Sjogren’s syndrome (pSS), rheumatoid arthritis (RA) and thyroid disease [12,22,23].

**Figure 1:**
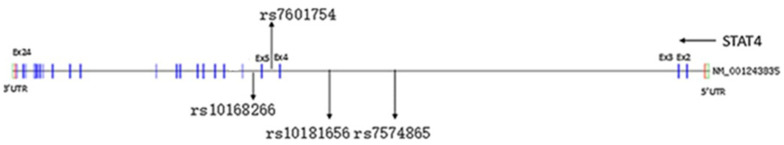
STAT4 SNPs location.

The four *STAT4* polymorphisms investigated in this study (rs10181656, rs7574865, rs7601754, rs10168266) are located at different sites on the chromosome (Figure 1). SNP rs7574865 and rs10181656 are located in intron 3, while rs7601754 and rs10168266 are located in intron 4 and intron 5, respectively [12]. STAT4 is activated by interleukin (IL)-12 and IL -23, which promotes the differentiation of CD40+ T cells into Th1 and Th17 cells and the production of interferon-γ (IFN-γ) and IL-17. The Th1 signaling pathway is considered to be the most important proinflammatory part of pathogenesis in multiple sclerosis (MS). In contrast, the Th17 signaling pathway is involved in the pathogenetic mechanisms of MS. It is well known that ON is closely related to MS, and it is often difficult to distinguish the specific symptoms of these diseases [24,25], so the SNPs studied in our work could have a direct impact on the manifestation of ON.

### 1.4. Optic Neuritis and Multiple Sclerosis

Multiple sclerosis is a chronic inflammatory, demyelinating disease of the central nervous system characterized by white matter damage and axonal loss. The causes of this disease are not yet fully understood, but it is often associated with viral infections or other autoimmune processes. The diagnosis is made on the basis of clinical symptoms or instrumental tests [24].

When optic neuritis and multiple sclerosis occur together, it is often difficult to distinguish the specific signs of these diseases, as ON can be part of the manifestations of multiple sclerosis [26]. In about 20% of MS patients, this disease first manifests as inflammation of the optic nerve; in about 50% of MS patients, inflammation of the optic nerve occurs during their lifetime [24,25].

## 2. Materials and Methods

### 2.1. Ethics Statement

To determine whether there was an association between *STAT4* and ON, we conducted a study involving subjects who had signed personal forms for approval by the Kaunas Regional Biomedical Research Ethics Committee (No. BE-2-102). 

### 2.2. Subjects

Subjects with ON were included according to the inclusion/diagnostic criteria (Table 1) [27]. 

Patients with other diseases of the optic nerve, systemic illnesses (diabetes mellitus, oncological diseases, systemic tissue disorders, chronic infectious diseases, conditions after organ or tissue transplantation), opacities of the optical system or because of poor quality of fundus photography were excluded [27,28].

The diagnosis of MS was made on the basis of consultation with the neurologist and MRI records. The neurological diagnosis of MS was established according to the revised and widely accepted McDonald criteria [29].

The control group comprised healthy individuals admitted to the Department of Ophthalmology at the Hospital of Lithuanian University of Health Sciences for routine ophthalmological examinations. Matching was performed based on the age and gender of patients diagnosed with optic neuritis (ON). Inclusion in the control group required participants to exhibit no ophthalmological eye disorders during the examination and to provide informed consent. Exclusion criteria encompassed any pre-existing eye disorders and the use of epileptic and sedative medications.

### 2.3. Genotyping

The analysis of *STAT4* gene polymorphisms, specifically rs10181656, rs7574865, rs7601754, and rs10168266, was conducted at the Laboratory of Ophthalmology, Neuroscience Institute, LUHS. Genotyping of *STAT4* polymorphisms was carried out using the real-time polymerase chain reaction (RT-PCR) method. The identification of all single-nucleotide polymorphisms was performed through TaqMan^®^ Genotyping assays (Applied Biosystems, New York, NY, USA; Thermo Fisher Scientific, Inc., Waltham, MA, USA), specifically using the assays: C__30530761_10, C__29882391_10, C__11515729_20, and C__29936344_10, following the manufacturer’s protocols on a StepOne Plus system (Applied Biosystems). The genotyping process utilized the “StepOnePlus” real-time PCR quantification system (Thermo Fisher Scientific, Singapore).

### 2.4. Serum IL-9 Levels Measurement

Serum STAT4 levels were measured in duplicate in control subjects and patients with ON. The determination was performed by enzyme-linked immunosorbent assay (ELISA) using the Signal Transducer And Activator Of Transcription 4 (STAT4) ELISA kit (Cat. No. abx156860), standard curve sensibility range: 0.312–20 ng/mL, sensitivity <0.12 ng/mL. Serum levels were analyzed according to the manufacturer’s instructions using a Multiskan FC Microplate Photometer (Thermo Scientific, Waltham, MA, USA/Canada) at 450 nm.

### 2.5. Statistical Analysis

Statistical data analysis for genotype and allele distribution and binary logistic regression was carried out with the program “IBM SPSS Statistics 29.0”, while haplotype analysis was performed with the online program “SNPStats”. Qualitative data are presented in absolute numbers and percentages. The hypothesis about the distribution of the quantitative data was tested using the Shapiro–Wilk test. The median was calculated for the test characteristics that did not fulfil the criteria of normal distribution.

After evaluating the age of the subjects and if the studied groups did not meet the criterion of normal distribution, the Mann–Whitney U test was used (to assess the difference between two independent groups). Binary logistic regression calculations were also performed, evaluating the influence of genotypes on the occurrence of ON, indicating the 95% likelihood ratio (OR) and confidence interval (CI).

In our study, we applied the Bonferroni correction because of multiple comparisons. Therefore, differences are considered statistically significant if the *p*-value is <0.05/4 (<0.0125). Only statistically significant results are presented in this article.

## 3. Results

During the study, subjects were divided into two groups. The first group consisted of subjects with optic neuritis (ON) with or without multiple sclerosis (*n* = 81). Of these, 28 were men (34.56%), 53 were women (65.44%), and the average age of the subjects was 33 years. Of these, 74 were examined for multiple sclerosis. The control group was composed of 158 subjects: 37 males (23.41%), 121 females (76.59%), and their average age was 29.5 years. The characteristics of the subjects are shown in Table S1 of the Supplementary Materials.

The genotypes and allele distributions of *STAT4* genes rs10181656, rs7574865, rs7601754, and rs10168266 were analysed in the ON group and compared with the control group. However, no statistically significant differences were found between the groups (Supplementary Materials, Table S2). Binary logistic regression also revealed no statistically significant differences between patients with optic neuritis and the control group (Supplementary Materials, Table S3).

*STAT4* rs10181656, rs7574865, rs7601754, rs10168266 genotype and allele distribution were compared by age group (≤30 and >30 years) and gender. Unfortunately, no statistically significant differences were found (Supplementary Material, Tables S4 and S5). Binary logistic regression between patients with optic neuritis and the control group also did not yield statistically significant results when divided by age groups (≤30 and >30 years) and gender (Supplementary Materials, Tables S6 and S7).

Another analysis was performed when subjects with ON were divided into two groups: with multiple sclerosis and without multiple sclerosis. Statistically significant differences were found when comparing ON with MS and the control group. The *STAT4* rs7574865 G allele was statistically significantly more frequent in the group of ON patients with MS than in the control group (84.38% vs. 65.93%, *p* = 0.003). In addition, *STAT4* rs10168266 C allele was statistically significantly more frequent in ON group of patients with MS than in control group (89.06% vs. 71.75%, *p* = 0.003). The results are shown in Table 2. However, binary logistic regression did not yield statistically significant results between patients with MS and without MS (Supplementary Materials, Table S8).

*STAT4* (rs10181656, rs7574865, rs7601754, rs10168266) haplotype analysis was performed. Pairwise linkage disequilibrium (LD) between studied polymorphisms was observed. The deviation between the predicted haplotype frequency and the observed frequency (D’) was calculated, and the square of the correlation coefficient (r^2^) was estimated. Data are presented in Table 3.

Haplotype frequencies and statistical analysis on the occurrence of ON have shown that individuals carrying *STAT4* rs10181656, rs7574865, rs7601754, rs10168266 haplotypes G-G-A-C and C-T-A-T were associated with 11.5- and 19.5-fold increased odds of ON (OR = 11.51; 95% CI: 2.29–57.80; *p* = 0.003; OR = 19.47; 95% CI: 2.25–168.17; *p* = 0.008, respectively) (Table 4).

In addition, we performed haplotype analysis in ON with and without MS groups vs. the control group. Statistical analysis of the incidence of ON without MS has shown that individuals carrying *STAT4* rs10181656, rs7574865, rs7601754, rs10168266 haplotypes C-T-A-T and G-G-A-C were respectively associated with 32.6- and 9-fold increased odds of ON without MS occurrence (OR = 32.55; 95% CI: 3.66–289.72; *p* = 0.002; OR = 9.05; 95% CI: 1.53–53.35; *p* = 0.016, respectively) (Table 5). Unfortunately, haplotype analysis of ON with MS vs. control group did not reveal any statistically significant results (Supplementary Materials, Table S9).

**Figure 2:**
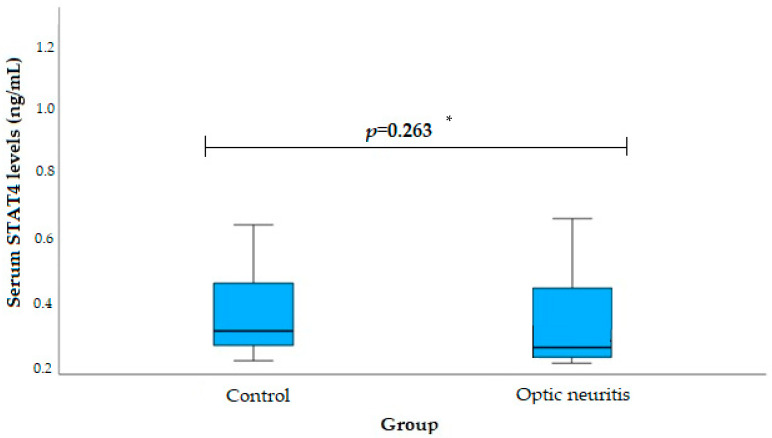
Serum STAT4 levels (ng/mL) in ON and control groups. * Mann–Whitney U test was used.

STAT4 serum levels in ON patients and control group subjects were evaluated. We found that STAT4 serum levels were not statistically significantly different between groups (0.290 (0.248) ng/mL vs. 0.314 (0.292) ng/mL, *p* = 0.263) (Figure 2).

## 4. Discussion

In this study, we investigated the associations of *STAT4* gene (rs10181656, rs7574865, rs7601754, rs10168266) polymorphisms and STAT4 serum level with ON. According to the scientific literature, the rs10181656, rs7574865, rs7601754 and rs10168266 polymorphisms influence the manifestation of various diseases, including autoimmune diseases. Associations have been found with Graves’ disease (GD) and Hashimoto’s thyroiditis (HT) as well as an increased risk of rheumatoid arthritis, systemic lupus erythematosus and type 1 diabetes [12,30,31,32,33,34]. However, no study has examined the impact of the occurrence of ON.

Hye-Soon Lee and co-authors conducted a study to determine the association of rs7574865, rs8179673, rs10181656 with type 1 diabetes. Patients were categorized into early- and late-period subgroups based on the time of diagnosis. Rs7574865, rs8179673 and rs10181656 showed statistically significant associations with diabetes type 1 in the early-onset subgroup (rs7574865, OR = 1.44 (1.03–2.01), *p* < 0.05), but not in the late-onset subgroup. This provides evidence that *STAT4* is not only disease-specific but also associated with early development of type 1 diabetes [23]. Yongsoo Park and co-authors also studied *STAT4* gene expression in type 1 CD. The results show an association between *STAT4* haplotype (rs11889341, rs7574865, rs8179673, and rs10181656) and type 1 diabetes and rheumatoid arthritis. Researchers have found that *STAT4* alleles and the same haplotypes can influence cytokine signaling and thus the development of autoimmune thyroid disease (AITD) and type 1 diabetes [35].

Based on the further aims of our work, an analysis of *STAT4* polymorphisms (rs10181656, rs7574865, rs7601754, rs10168266) was performed to determine associations with ON in subjects with ON and control groups as a function of subject age. We did not obtain statistically significant data (*p* < 0.0125). Bi C and co-authors conducted a case-control study in a Han population in northeastern China. Two SNPs in the *STAT4* gene and their association with type 1 diabetes were investigated. This disease is associated with autoimmune body lesions such as ON. The results of this study showed that one of the two SNPs studied (rs7574865) was strongly associated with type 1 diabetes in a northeast Chinese population compared with healthy controls (*p* < 0.05). Another SNP (rs3024866) showed a weak association with the onset of type 1 diabetes, but when the researchers stratified patients by age of onset, the alleles of all four single nucleotide polymorphisms and the same haplotypes showed a significant association with susceptibility to type 1 diabetes in the early-onset subgroup (*p* < 0.01) and not in the late-onset subgroup [22].

STAT4 is a central mediator in the development of inflammation during protective immune responses and immune-mediated disease [36]. Rheumatoid arthritis (RA) is a chronic systemic inflammatory disease characterized by articular and extra-articular manifestations, including cardiovascular disease [37]. Both genetic and environmental factors have been reported to influence the pathogenesis of RA. STAT4 contributes to the differentiation and proliferation of Th1 and Th17 cells, which play a critical role in chronic inflammatory diseases [38]. Researchers found that increased expression of STAT4 protein in dendritic cells in the synovial membrane is associated with serum rheumatoid factor, which is a risk factor for RA [39,40]. Diabetic retinopathy (DR) is considered one of the most important microvascular complications of diabetes, usually resulting from moderate to severe vision loss [41]. One of the main reasons for this irreversible visual impairment is retinal neovascularization. DR is usually associated with dysfunction of signaling pathways and abnormal expression of functional molecules [42]. Jun Shao and co-authors conducted a case-control study in China, experimenting with certified hRECs cells. The results of this study showed that miR-223-3p plays a key role in the development of DR and that the STAT4 protein recognizes mir223-3p as a direct target and can enhance the expression of mir223-3p. This study revealed a new potential signaling pathway in the progression of DR [43].

The study’s main limitation is its small sample size due to the rarity of ON. Future studies should prioritize increasing the number of subjects to enhance result accuracy. A larger sample size offers advantages such as increased trait variation, higher significance levels, and lower measurement error [44]. These improvements bolster the study’s reliability and its potential to inform further exploration of ON pathology, contributing to strategies for preventing rapid disease progression. Addressing this limitation remains a priority in future research efforts, emphasizing our commitment to advancing ON understanding. The results of the research conducted can be used to further study this pathology and prevent the rapid progression of the disease. Considering the crucial roles of *STAT4* in inflammation and autoimmunity, targeting this gene may offer a novel avenue for therapeutic intervention in ON, especially in the context of MS [45]. The molecular insights provided by our study contribute to understanding the underlying mechanisms of ON and MS pathogenesis. This understanding, combined with the known efficacy of current immunosuppressive treatments in modulating these processes, opens up possibilities for developing targeted and personalized therapeutic options. Therefore, the performed scientific researches contribute to establishing a relationship between *STAT4* gene SNP (rs10181656, rs7574865, rs7601754, rs10168266) and autoimmune diseases. With a larger sample size, it is worthwhile to search for other associations of *STAT4* harboring other SNPs with ON.

## 5. Conclusions

In conclusion, the G-G-A-C and C-T-A-T haplotypes of the STAT4 gene, represented by rs10181656, rs7574865, rs7601754, and rs10168266, exhibit significant associations with ON occurrences. Specifically, these haplotypes are linked to an 11.5- and 19.5-fold increased odds of ON, both with *p*-values of 0.003 and 0.008, respectively. Moreover, when considering cases of optic neuritis without MS, the same STAT4 haplotypes, G-G-A-C and C-T-A-T, demonstrate notable associations with 32.6- and 9-fold increased odds, supported by *p*-values of 0.002 and 0.016, respectively. Collectively, the current evidence suggests a potential risk role of the STAT4 G-G-A-C and C-T-A-T haplotypes in the context of both ON and ON without MS.

## Figures and Tables

**Table 1:** Optic neuritis (ON) inclusion/diagnostic criteria [27].

Symptoms	Typical ON
Age	Young patient < 50 years
Visual acuity loss time	Acute/subacute visual acuity loss
Visual acuity loss progression	Visual acuity loss progressing for few days or few weeks
Damage	Mostly one eye
Visual acuity	↓ in 90% of cases
Visual field	Changes noticed in 97% of cases
Color vision	In acute period, blue-yellow color vision loss; in subacute period, red-green color vision loss
Visual evoked potentials (VEP)	↓ VEP latency
Optical coherence tomography (OCT)	Optic nerve disc edema (mostly in superior and nasal quadrants), noticed in 20% of patients
Pain	Acute painful visual acuity loss, especially ↑ with eye movement
Optic nerve disc	Mostly normal optic nerve disc
Vitreous	Normal
Orbit	Normal
Anamnesis	ON in anamnesis or MS in anamnesis. Patients without MS had MS-like lesions but were not followed up after ON treatment in our study, only redirected for neurological follow-up.
Neurological symptoms	Neurological symptoms, allowing to suspect MS
Treatment effect using steroids	Shortens the duration of the disease
Improvement	Spontaneous improvement in 2–3 weeks
Prognosis	Mostly good
Recurrence (5–10 years)	28%

**Table 2:** Distribution of *STAT4* (rs10181656, rs7574865, rs7601754, rs10168266) genotypes, optic nerve inflammation groups in patients with and without multiple sclerosis.

Gene	Genotype	With MS			Without MS		
		ON Group (*n* = 32) *n* (%)	Control Group (*n* = 158) *n* (%)	*p*-Value	ON Group (*n* = 42) *n* (%)	Control Group (*n* = 158) *n* (%)	*p*-Value
*STAT4* (rs10181656)	CC	20 (62.5)	90 (56.96)	0.721	26 (61.9)	90 (56.96)	0.827
	CG	11 (34.38)	58 (36.71)		14 (33.33)	58 (36.71)	
	GG	1 (3.13)	10 (6.33)		2 (4.76)	10 (6.33)	
	Allele:			0.024			0.020
	C	51 (79.69)	238 (65.38)		66 (78.57)	238 (65.38)	
	G	13 (20.31)	126 (34.62)		18 (21.43)	126 (34.62)	
*STAT4* (rs7574865)	GG	23 (71.88)	91 (57.59)	0.303	25 (59.52)	91 (57.59)	0.968
	GT	8 (25)	56 (35.44)		14 (33.33)	56 (35.44)	
	TT	1 (3.13)	11 (6.96)		3 (7.14)	11 (6.96)	
	Allele:			0.003			0.070
	G	54 (84.38)	238 (65.93)		64 (76.19)	238 (65.93)	
	T	10 (15.63)	123 (34.07)		20 (23.81)	123 (34.07)	
*STAT4* (rs7601754)	AA	22 (68.75)	121 (76.58)	0.631	31 (73.81)	121 (76.58)	0.927
	GA	9 (28.13)	34 (21.52)		10 (23.81)	34 (21.52)	
	GG	1 (3.13)	3 (1.9)		1 (2.38)	3 (1.9)	
	Allele:			0.547			0.198
	A	53 (82.81)	276 (79.54)		72 (85.71)	276 (79.54)	
	G	11 (17.19)	71 (20.46)		12 (14.29)	71 (20.46)	
*STAT4* (rs10168266)	CC	26 (81.25)	105 (66.46)	0.213	27 (64.29)	105 (66.46)	0.873
	CT	5 (15.63)	49 (31.01)		13 (30.95)	49 (31.01)	
	TT	1 (3.13)	4 (2.53)		2 (4.76)	4 (2.53)	
	Allele:			0.003			0.135
	C	57 (89.06)	259 (71.75)		67 (79.76)	259 (71.75)	
	T	7 (10.94)	102 (28.25)		17 (20.24)	102 (28.25)	

*p*-value—significance level; After Bonferroni correction, differences were considered statistically significant when *p* < 0.0125.

**Table 3:** Linkage disequilibrium between studied polymorphisms in patients with optical neuritis and control group.

SNPs	ON vs. Controls		
	D’	r^2^	*p-*Value
rs10181656–rs7574865	0.867	0.743	<0.001
rs10181656–rs7601754	0.858	0.034	<0.001
rs10181656–rs10168266	0.707	0.348	<0.001
rs7574865–rs7601754	0.998	0.046	<0.001
rs7574865–rs10168266	0.814	0.467	<0.001
rs7601754–rs10168266	0.998	0.032	<0.001

D’: the deviation between the expected haplotype frequency and the observed frequency; r^2^: the square of the haplotype frequency correlation coefficient.

**Table 4:** Haplotype association with the predisposition to optical neuritis occurrence.

Haplotype	*STAT4* rs10181656	*STAT4* rs7574865	*STAT4* rs7601754	*STAT4* rs10168266	Frequency		OR (95% CI)	*p-*Value
					Control	ON		
1	C	G	A	C	59.43	58.29	1.00	–
2	G	T	A	T	14.57	11.01	0.55 (0.27–1.09)	0.087
3	C	G	G	C	11.78	14.81	1.24 (0.69–2.24)	0.470
4	G	T	A	C	9.16	4.15	0.47 (0.19–1.17)	0.100
5	C	G	A	T	2.59	0.70	0.29 (0.05–1.62)	0.160
6	G	G	A	C	0.64	5.17	11.51 (2.29–57.80)	0.003
7	C	T	A	T	0.32	4.30	19.47 (2.25–168.17)	0.008
rare	*	*	*	*	NA	NA	0.69 (0.06–7.49)	0.760

OR: odds ratio; CI: confidence interval; *p*-value: significance level (after Bonferroni correction statistically significant when *p* < 0.0125). * rare—polymorphic alleles with <1% frequency; NA—not applicable.

**Table 5:** Haplotype association with the predisposition to optical neuritis without MS occurrence.

Haplotype	*STAT4* rs10181656	*STAT4* rs7574865	*STAT4* rs7601754	*STAT4* rs10168266	Frequency		OR (95% CI)	*p-*Value
					Control	ON		
1	C	G	A	C	59.43	55.58	1.00	–
2	G	T	A	T	14.57	11.52	0.57 (0.24–1.38)	0.210
3	C	G	G	C	11.78	14.28	1.21 (0.57–2.61)	0.620
4	G	T	A	C	9.16	4.81	0.50 (0.15–1.64)	0.260
5	C	G	A	T	2.59	1.24	0.49 (0.06–4.41)	0.530
6	C	T	A	T	0.32	7.47	32.55 (3.66–289.72)	0.002
7	G	G	A	C	0.64	5.09	9.05 (1.53–53.35)	0.016

OR: odds ratio; CI: confidence interval; *p*-value: significance level (after Bonferroni correction statistically significant when *p* < 0.0125).

## Data Availability

Data is contained within the article and Supplementary Materials.

## Supplementary Materials

The following supporting information can be downloaded at: https://www.mdpi.com/article/10.3390/jcm13010010/s1, Table S1: Characteristics; Table S2: Distribution of *STAT4* rs10181656, rs7574865, rs7601754, rs10168266 genotypes in patients with optic neuritis and controls; Table S3: STAT4 (rs10181656, rs7574865, rs7601754, rs10168266) binary logistic regression analysis of genotypes; Table S4: *STAT4* (rs10181656, rs7574865, rs7601754, rs10168266) distribution of genotypes in patients with optic neuritis and controls according to the age of the subjects; Table S5: Distribution of *STAT4* (rs10181656, rs7574865, rs7601754, rs10168266) genotypes in patients with optic neuritis and controls according to gender; Table S6: Binary logistic regression analysis of *STAT4* (rs10181656, rs7574865, rs7601754, rs10168266) genotypes by age of the subjects; Table S7: Binary logistic regression analysis of *STAT4* (rs10181656, rs7574865, rs7601754, rs10168266) genotypes by gender; Table S8: Binary logistic regression analysis of STAT4 (rs10181656, rs7574865, rs7601754, rs10168266) genotypes in patients with multiple sclerosis (with MS) and without multiple sclerosis (without MS); Table S9: Haplotype association with the predisposition to optical neuritis with MS occurrence.
