# Supplementary material for: Optic Neuritis: The Influence of Gene Polymorphisms and Serum Levels of STAT4 (rs10181656, rs7574865, rs7601754, rs10168266)
Source: J Clin Med. 2023 Dec 19;13(1):10. doi: 10.3390/jcm13010010 (PMC10779575; doi:10.3390/jcm13010010)
Supplement: Supplementary file 1 [file jcm-13-00010-s001.zip › jcm-2704674-supplementary.pdf]

Supplementary Materials: The following supporting information can be downloaded at: www.mdpi.com/xxx/s1, Table S1: Characteristics; Table S2: Distribution of *STAT4* rs10181656, rs7574865, rs7601754, rs10168266 genotypes in patients with optic neuritis and controls; Table S3: STAT4 (rs10181656, rs7574865, rs7601754, rs10168266) binary logistic regression analysis of genotypes; Table S4: *STAT4* (rs10181656, rs7574865, rs7601754, rs10168266) distribution of genotypes in patients with optic neuritis and controls according to the age of the subjects; Table S5: Distribution of *STAT4* (rs10181656, rs7574865, rs7601754, rs10168266) genotypes in patients with optic neuritis and controls according to gender; Table S6: Binary logistic regression analysis of *STAT4* (rs10181656, rs7574865, rs7601754, rs10168266) genotypes by age of the subjects; Table S7: Binary logistic regression analysis of *STAT4* (rs10181656, rs7574865, rs7601754, rs10168266) genotypes by gender; Table S8: Binary logistic regression analysis of STAT4 (rs10181656, rs7574865, rs7601754, rs10168266) genotypes in patients with multiple sclerosis (with MS) and without multiple sclerosis (without MS); Table S9: Haplotype association with the predisposition to optical neuritis with MS occurrence;

Table S1. Characteristics.

**Characteristics**	**Group**		**p-value**
	**ON group (n=81)**	**Control group (n=158)**	
Male	28 (34.56%)	37 (23.41%)	0.067
Female	53 (65.44%)	121 (76.59%)	
Age median (IQR)	33 (16.5)	29.5 (14)	0.325
Multiple sclerosis:<br>-With<br>-Without<br>-NA	 42 (51.85%<br>32 (39.5%)<br>7 (8.64%)	-	-

IQR – interquartile range

Table S2. Distribution of *STAT4* rs10181656, rs7574865, rs7601754, rs10168266 genotypes in patients with optic neuritis and controls.

**Gene**	**Genotype/Allele**	**ON group (n=81) N (%)**	**Control group (n=158) N (%)**	**p-value**	**P-value HWE**
***STAT4* (rs10181656)**	CC	50 (61.73)	90 (56.96)	0.621	0.950
	CG	28 (34.57)	58 (36.71)		
	GG	3 (3.70)	10 (6.33)		
	In total:	81 (100)	158 (100)		
	Allele:<br>C<br>G	128 (79.01)<br>34 (20.99)	238 (75.32)<br>78 (24.68)	0.366	
***STAT4* (rs7574865)**	GG	52 (64.2)	91 (57.6)	0.585	0.677
	GT	25 (30.86)	56 (35.44)		
	TT	4 (4.94)	11 (6.96)		
	In total:	81 (100)	158 (100)		
	Allele:<br>G<br>T	129 (79.63)<br>33 (20.37)	238 (75.32)<br>78 (24.68)	0.751	
***STAT4* (rs7601754)**	AA	59 (72.84)	121 (76.58)	0.810	0.736
	GA	20 (24.69)	34 (21.52)		
	GG	2 (2.47)	3 (1.9)		
	In total:	81 (100)	157 (100)		

	Allele:<br>A<br>G	 138 (85.19)<br>24 (14.81)	 276 (87.34)<br>40 (12.66)	0.511	
STAT4 **(rs10168266)**	CC	57 (70.37)	105 (66.46)	0.655	0.539
	CT	21 (25.93)	49 (31.01)		
	TT	3 (3.70)	4 (2.53)		
	In total:	81 (100)	158 (100)		
	Allele:<br>C<br>T	 135 (83.33)<br>27 (16.67)	 259 (81.96)<br>57 (18.04)	0.709	

p-value – level of significance; p-value HWE - significance level according to the Hardy-Weinberg equilibrium, differences were considered statistically significant when p<0.05.

Table S3. *STAT4* (rs10181656, rs7574865, rs7601754, rs10168266) binary logistic regression analysis of genotypes.

STAT4 **(rs10181656):**				
**Model**	**Genotype/Allele**	**OR (95% CI)**	**p-value**	**AIC**
Codominant	CG *vs.* CC<br>GG *vs.* CC	0.869 (0.492-1.534)<br>0.540 (0.142-2.054)	0.628<br>0.366	309.070
Dominant	CG+GG *vs.* CC	0.821 (0.474-1.419)	0.479	307.565
Recessive	GG *vs.* CC+CG	0.569 (0.152-2.129)	0.402	307.306
Overdominant	CG *vs.* CC+GG	0.911 (0.52-1.562)	0.744	307.962
Additive	C	0.810 (0.512-1.28)	0.367	307.240
STAT4 **(rs7574865):**				
**Model**	**Genotype/Allele**	**OR (95% CI)**	**p-value**	**AIC**
Codominant	GT *vs.* GG<br>TT *vs.* GG	0.781 (0.437-1.398)<br>0.638 (0.193-2.1)	0.406<br>0.458	308.983
Dominant	GT+TT *vs.* GG	0.757 (0.436-1.317)	0.325	307.091
Recessive	TT *vs.* GG+GT	0.694 (0.214-2.253)	0.543	307.682
Overdominant	GT *vs.* GG+TT	0.813 (0.458-1.442)	0.479	307.564
Additive	G	0.789 (0.503-1.239)	0.303	306.984
STAT4 **(rs7601754):**				
**Model**	**Genotype/Allele**	**OR (95% CI)**	**p-value**	**AIC**
Codominant	GA *vs.* AA<br>GG *vs.* AA	1.206 (0.64-2.274)<br>1.367 (0.222-8.405)	0.562<br>0.736	309.652
Dominant	GA+GG *vs.* AA	1.219 (0.661-2.25)	0.526	308.669
Recessive	GG *vs.* AA+GA	1.308 (0.214-7.989)	0.771	307.986
Overdominant	GA *vs.* AA+GG	1.196 (0.636-2.249)	0.579	307.764
Additive	A	1.195 (0.697-2.049)	0.518	307.656
STAT4 **(rs10168266*)***				
**Model**	**Genotype/Allele**	**OR (95% CI)**	**p-value**	**AIC**
Codominant	CT *vs.* CC<br>TT *vs.* CC	0.789 (0.431-1.445)<br>1.382 (0.299-6.389)	0.443<br>0.679	309.223
Dominant	CT+TT *vs.* CC	0.834 (0.467-1.49)	0.540	307.690
Recessive	TT *vs.* CC+CT	1.481 (0.323- 6.781)	0.613	307.819
Overdominant	CT *vs.* CC+CT	0.779 (0.427-1.419)	0.414	307.392
Additive	C	0.908 (0.547-1.506)	0.708	307.927

p-value – level of significance; differences were considered statistically significant when p<0.05, OR – capability ratio; AIC – Akaike.

Table S4. *STAT4* (rs10181656, rs7574865, rs7601754, rs10168266) distribution of genotypes in patients with optic neuritis and controls according to the age of the subjects.

Gene	Genotype	≤ 30 y.		P-value	>30 y.		P-value
		ON group(n =38) N (%)	Control group (n=88) N (%)		ON group (n=43) N (%)	Control group (n=70) N (%)	
***STAT4* (rs10181656)**	CC	24 (63.16)	46 (52.27)	0.425	26 (60.47)	44 (62.86)	0.915
	CG	13 (34.2)	36 (40.91)		15 (34.88)	22 (31.43)	
	GG	1 (2.63)	6 (6.82)		2 (4.65)	4 (5.71)	
	Allele: C<br>G	61 (80.26)<br>15 (19.74)	128 (72.73)<br>48 (27.27)	0.205	67 (77.91)<br>19 (22.09)	110 (78.57)<br>30 (21.43)	0.906
***STAT4* (rs7574865)**	GG	25 (65.79)	47 (53.41)	0.432	27 (62.79)	44 (62.86)	0.967
	GT	11 (28.95)	34 (38.64)		14 (32.56)	22 (31.43)	
	TT	2 (5.26)	7 (7.95)		2 (4.65)	4 (5.71)	
	Allele: G<br>T	61 (80.26)<br>15 (19.74)	128 (72.73)<br>48 (27.27)	0.205	68 (79.07)<br>18 (20.93)	110 (78.57)<br>30 (21.43)	0.929
***STAT4* (rs7601754)**	AA	25 (65.79)	66 (75.0)	0.564	34 (79.07)	55 (78.57)	0.929
	GA	12 (31.58)	20 (22.73)		8 (18.60)	14 (20.0)	
	GG	1 (2.63)	2 (2.27)		1 (2.33)	1 (1.43)	
	Allele: A<br>G	62 (81.58)<br>14 (18.42)	152 (86.36)<br>24 (13.64)	0.330	76 (88.37)<br>10 (11.63)	124 (88.57)<br>16 (11.43)	0.964
***STAT4* (rs10168266)**	CC	28 (73.68)	55 (62.5)	0.328	29 (67.44)	50 (71.43)	0.873
	CT	8 (21.05)	30 (34.09)		13 (30.23)	19 (27.14)	
	TT	2 (5.26)	3 (3.41)		1 (2.33)	1 (1.43)	
	Allele: C<br>T	64 (84.2)<br>12 (15.79)	140 (79.55)<br>36 (20.45)	0.387	71 (82.56)<br>15 (17.44)	119 (85.0)<br>21 (15.0)	0.626

p-value – level of significance; differences were considered statistically significant when p<0.05.

Table S5. Distribution of *STAT4* (rs10181656, rs7574865, rs7601754, rs10168266) genotypes in patients with optic neuritis and controls according to gender.

Gene	Genotype	Males			Females		
		ON group (n=28) N (%)	Control group (n=37) N (%)	P-value	ON group (n=53) N (%)	Control group (n=121) N (%)	P-value
***STAT4* (rs10181656)**	CC	20 (71.43)	23 (62.16)	0.647	30 (56.60)	67 (55.37)	0.858
	CG	7 (25)	11 (29.73)		21 (39.62)	47 (38.84)	
	GG	1 (3.57)	3 (8.11)		2 (3.77)	7 (5.79)	
	Allele:			0.329			0.747

	C<br>G	47 (83.93)<br>9 (16.07)	57 (77.03)<br>17 (22.97)		81 (76.42)<br>25 (23.58)	181 (74.79)<br>61 (25.21)	
***STAT4* (rs7574865)**	GG	20 (71.43)	25 (67.57)	0.945	32 (60.38)	66 (54.55)	0.661
	GT	6 (21.43)	9 (24.32)		19 (35.85)	47 (38.84)	
	TT	2 (7.14)	3 (8.11)		2 (3.77)	8 (6.61)	
	Allele:<br>G<br>T	 46 (82.14)<br>10 (17.86)	 59 (79.73)<br>15 (20.27)	0.729	 83 (78.30)<br>23 (21.7)	 179 (73.97)<br>63 (26.03)	0.388
***STAT4* (rs7601754)**	AA	19 (67.86)	29 (78.38)	0.475	40 (75.47)	92 (76.03)	0.832
	GA	8 (28.57)	6 (16.22)		12 (22.64)	28 (23.14)	
	GG	1 (3.57)	2 (5.41)		1 (1.89)	1 (0.83)	
	Allele:<br>A<br>G	 46 (82.14)<br>10 (17.86)	 64 (86.49)<br>10 (13.51)	0.496	 92 (86.8)<br>14 (13.21)	 212 (87.6)<br>30 (12.4)	0.833
***STAT4* (rs10168266)**	CC	21 (75)	27 (72.97)	0.606	36 (67.92)	78 (64.46)	0.896
	CT	5 (17.86)	9 (24.32)		16 (30.2)	40 (33.06)	
	TT	2 (7.14)	1 (2.70)		1 (1.89)	3 (2.48)	
	Allele:<br>C<br>T	 47 (83.93)<br>9 (16.07)	 63 (85.14)<br>11 (14.86)	0.849	 88 (83.02)<br>18 (16.98)	 196 (80.99)<br>46 (19.01)	0.653

p-value - level of significance; differences were considered statistically significant when $p<0.05$.

Table S6. Binary logistic regression analysis of *STAT4* (rs10181656, rs7574865, rs7601754, rs10168266) genotypes by age of the subjects.

**Age ≤30 years old**				
***STAT4* (rs10181656):**				
**Model**	**Genotype/Allele**	**OR (95% CI)**	**p-value**	**AIC**
Codominant	CG *vs.* CC<br>GG *vs.* CC	0.841 (0.409-1.73)<br>0.491 (0.097-2.473)	0.638<br>0.388	189.610
Dominant	CG+GG *vs.* CC	0.785 (0.392-1.574)	0.496	188.056
Recessive	GG *vs.* CC+CG	0.528 (0.108-2.587)	0.431	187.832
Overdominant	CG *vs.* CC+GG	0.901 (0.444-1.829)	0.774	188.440
Additive	C	0.775 (0.436-1.376)	0.384	187.748
***STAT4* (rs7574865):**				
**Model**	**Genotype/Allele**	**OR (95% CI)**	**p-value**	**AIC**
Codominant	GT *vs.* GG<br>TT *vs.* GG	0.888 (0.427-1.847)<br>0.679 (0.17-2.714)	0.750<br>0.679	190.168
Dominant	GT+TT *vs.* GG	0.849 (0.423-1.702)	0.644	188.309
Recessive	TT *vs.* GG+GT	0.712 (0.184-2.76)	0.623	188.270
Overdominant	GT *vs.* GG+TT	0.930 (0.454-1.903)	0.842	188.483
Additive	G	0.853 (0.492-1.477)	0.570	188.195
***STAT4* (rs7601754):**				
**Model**	**Genotype/Allele**	**OR (95% CI)**	**p-value**	**AIC**
Codominant	GA *vs.* AA<br>GG *vs.* AA	1.501 (0.673-3.364)<br>1.212 (0.106-13.830)	0.321<br>0.877	189.549
Dominant	GA+GG *vs.* AA	1.476 (0.678-3.214)	0.327	187.577

Recessive	GG *vs.* AA+GA	1.098 (0.097-12.416)	0.940	188.517
Overdominant	GA *vs.* AA+GG	1.493 (0.672-3.319)	0.325	187.572
Additive	A	1.367 (0.687-2.722)	0.373	187.743
STAT4 **(rs10168266):**				
**Model**	**Genotype/Allele**	**OR (95% CI)**	**p-value**	**AIC**
Codominant	CT *vs.* CC<br>TT *vs.* CC	0.754 (0.351-1.62)<br>1.354 (0.215-8.514)	0.470<br>0.747	189.819
Dominant	CT+TT *vs.* CC	0.802 (0.385-1.668)	0.554	188.169
Recessive	TT *vs.* CC+CT	1.481 (0.239-9.175)	0.673	188.349
Overdominant	CT *vs.* CC+CT	0.743 (0.348-1.585)	0.442	187.921
Additive	C	0.886 (0.469-1.672)	0.708	188.381
**Age >30 years old**				
STAT4 **(rs10181656):**				
**Model**	**Genotype/Allele**	**OR (95% CI)**	**p-value**	**AIC**
Codominant	CG *vs.* CC<br>GG *vs.* CC	1.005 (0.39-2.59)<br>0.804 (0.069-9.379)	0.991<br>0.862	122.346
Dominant	CG+GG *vs.* CC	0.983 (0.395-2.45)	0.971	120.377
Recessive	GG *vs.* CC+CG	0.803 (0.07-9.208)	0.860	120.346
Overdominant	CG *vs.* CC+GG	1.016 (0.397-2.599)	0.974	120.377
Additive	C	0.965 (0.44-2.117)	0.928	120.370
STAT4 **(rs7574865):**				
**Model**	**Genotype/Allele**	**OR (95% CI)**	**p-value**	**AIC**
Codominant	GT *vs.* GG<br>TT *vs.* GG	0.678 (0.254-1.811)<br>0.720 (0.062-8.378)	0.438<br>0.793	121.732
Dominant	GT+TT *vs.* GG	0.682 (0.266-1.752)	0.427	119.734
Recessive	TT *vs.* GG+GT	0.803 (0.7-9.208)	0.860	120.364
Overdominant	GT *vs.* GG+TT	0.688 (0.259-1.828)	0.453	119.803
Additive	G	0.735 (0.325-1.664)	0.461	119.818
STAT4 **(rs7601754):**				
**Model**	**Genotype/Allele**	**OR (95% CI)**	**p-value**	**AIC**
Codominant	GA *vs.* AA<br>GG *vs.* AA	0.849 (0.3-2.407)<br>1.577 (0.94-26.324)	0.758<br>0.751	122.165
Dominant	GA+GG *vs.* AA	0.901 (0.332-2.445)	0.838	120.336
Recessive	GG *vs.* AA+GA	1.636 (0.099-27.056)	0.731	120.261
Overdominant	GA *vs.* AA+GG	0.838 (0.297-2.366)	0.738	120.265
Additive	A	0.967 (0.404-2.314)	0.940	120.373
STAT4 **(rs10168266):**				
**Model**	**Genotype/Allele**	**OR (95% CI)**	**p-value**	**AIC**
Codominant	CT *vs.* CC<br>TT *vs.* CC	0.914 (0.336-2.491)<br>1.600 (0.096-26.749)	0.744	122.230
Dominant	CT+TT *vs.* CC	0.960 (0.365-2.522)	0.934	120.371
Recessive	TT *vs.* CC+CT	1.636 (0.99-27.056)	0.731	120.261
Overdominant	CT *vs.* CC+CT	0.901 (0.332-2.445)	0.838	120.336
Additive	C	1.013 (0.432-2.373)	0.977	120.377

p-value – level of significance; differences were considered as statistically significant when $p<0.05$, OR – capability ratio; AIC – Akaike.

Table S7. Binary logistic regression analysis of *STAT4* (rs10181656, rs7574865,

rs7601754, rs10168266) genotypes by gender.

**Males**				
***STAT4* (rs10181656):**				
**Model**	**Genotype/Allele**	**OR (95% CI)**	**p-value**	**AIC**
Codominant	CG *vs.* CC<br>GG *vs.* CC	0.732 (0.238-2.246)<br>0.383 (0.037-3.984)	0.585<br>0.422	91.957
Dominant	CG+GG *vs.* CC	0.657 (0.229-1.888)	0.436	90.242
Recessive	GG *vs.* CC+CG	0.420 (0.041-4.266)	0.463	90.258
Overdominant	CG *vs.* CC+GG	0.788 (0.26-2.387)	0.673	90.680
Additive	C	0.674 (0.288-1.579)	0.364	90.005
***STAT4* (rs7574865):**				
**Model**	**Genotype/Allele**	**OR (95% CI)**	**p-value**	**AIC**
Codominant	GT *vs.* GG<br>TT *vs.* GG	0.833 (0.254-2.735)<br>0.833 (0.127-5.479)	0.764<br>0.850	92.747
Dominant	GT+TT *vs.* GG	0.833 (0.286-2.431)	0.739	90.747
Recessive	TT *vs.* GG+GT	0.872 (0.136-5.604)	0.885	90.836
Overdominant	GT *vs.* GG+TT	0.848 (0.262-2.745)	0.784	90.783
Additive	G	0.883 (0.399-1.952)	0.758	90.763
***STAT4* (rs7601754):**				
**Model**	**Genotype/Allele**	**OR (95% CI)**	**p-value**	**AIC**
Codominant	GA *vs.* AA<br>GG *vs.* AA	2.035 (0.609-6.799)<br>0.763 (0.065-9.015)	0.248<br>0.830	91.384
Dominant	GA+GG *vs.* AA	1.717 (0.513-5.233)	0.342	89.952
Recessive	GG *vs.* AA+GA	0.648 (0.056-7.529)	0.729	90.734
Overdominant	GA *vs.* AA+GG	2.067 (0.623-6.851)	0.235	89.431
Additive	A	1.326 (0.547-3.215)	0.532	90.468
***STAT4* (rs10168266):**				
**Model**	**Genotype/Allele**	**OR (95% CI)**	**p-value**	**AIC**
Codominant	CT *vs.* CC<br>TT *vs.* CC	0.714 (0.208-2.451)<br>2.571 (0.218-30.318)	0.593<br>0.453	91.858
Dominant	CT+TT *vs.* CC	0.9 (0.293-2.763)	0.854	90.825
Recessive	TT *vs.* CC+CT	2.769 (0.238-32.184)	0.416	90.149
Overdominant	CT *vs.* CC+CT	0.676 (0.199-2.301)	0.531	90.459
Additive	C	1.082 (0.446-2.622)	0.862	90.829
**Females**				
***STAT4* (rs10181656):**				
**Model**	**Genotype/Allele**	**OR (95% CI)**	**p-value**	**AIC**
Codominant	CG *vs.* CC<br>GG *vs.* CC	0.998 (0.51-1.952)<br>0.638 (0.125-3.255)	0.995<br>0.589	217.597
Dominant	CG+GG *vs.* CC	0.951 (0.469-1.824)	0.880	215.896
Recessive	GG *vs.* CC+CG	0.639 (0.128-3.182)	0.584	215.597
Overdominant	CG *vs.* CC+GG	1.033 (0.534-2.001)	0.923	215.910
Additive	C	0.912 (0.527-1.577)	0.741	215.809
***STAT4* (rs7574865):**				
**Model**	**Genotype/Allele**	**OR (95% CI)**	**p-value**	**AIC**
Codominant	GT *vs.* GG<br>TT *vs.* GG	0.834 (0.422-1.645)<br>0.516 (0.103-2.569)	0.600<br>0.419	217.052
Dominant	GT+TT *vs.* GG	0.788 (0.408-1.518)	0.476	215.407

Recessive	TT *vs.* GG+GT	0.554 (0.114-2.701)	0.465	215.328
Overdominant	GT *vs.* GG+TT	0.880 (0.45-1.719)	0.708	215.778
Additive	G	0.783 (0.452-1.358)	0.385	215.146
***STAT4* (rs7601754):**				
**Model**	**Genotype/Allele**	**OR (95% CI)**	**p-value**	**AIC**
Codominant	GA *vs.* AA<br>GG *vs.* AA	0.986 (0.456-2.132)<br>2.300 (0.14-37.693)	0.971<br>0.559	217.582
Dominant	GA+GG *vs.* AA	1.031 (0.486-2.187)	0.937	215.913
Recessive	GG *vs.* AA+GA	2.308 (0.142-37.603)	0.557	215.583
Overdominant	GA *vs.* AA+GG	0.972 (0.45-2.099)	0.943	215.914
Additive	A	1.079 (0.539-2.161)	0.831	215.874
***STAT4* (rs10168266):**				
**Model**	**Genotype/Allele**	**OR (95% CI)**	**p-value**	**AIC**
Codominant	CT *vs.* CC<br>TT *vs.* CC	0.876 (0.43-1.748)<br>0.722 (0.073-7.185)	0.689<br>0.781	217.698
Dominant	CT+TT *vs.* CC	0.857 (0.431-1.702)	0.659	215.722
Recessive	TT *vs.* CC+CT	0.756 (0.077-7.444)	0.811	215.859
Overdominant	CT *vs.* CC+CT	0.876 (0.436-1.76)	0.709	215.779
Additive	C	0.862 (0.463-1.607)	0.641	215.699

p-value – level of significance; differences were considered as statistically significant when $p<0.05$, OR – capability ratio; AIC – Akaike.

Table S8. Binary logistic regression analysis of *STAT4* (rs10181656, rs7574865, rs7601754, rs10168266) genotypes in patients with multiple sclerosis (with MS) and without multiple sclerosis (without MS).

**With MS**				
***STAT4* (rs10181656):**				
**Model**	**Genotype/Allele**	**OR (95% CI)**	**p-value**	**AIC**
Codominant	CG *vs.* CC<br>GG *vs.* CC	0.853 (0.381-1.911)<br>0.450 (0.054-3.719)	0.700<br>0.459	175.554
Dominant	CG+GG *vs.* CC	0.794 (0.363-1.736)	0.563	173.944
Recessive	GG *vs.* CC+CG	0.477 (0.059-3.867)	0.488	173.704
Overdominant	CG *vs.* CC+GG	0.903 (0.407-2.006)	0.802	174.219
Additive	C	0.778 (0.402-1.507)	0.457	173.708
***STAT4* (rs7574865):**				
**Model**	**Genotype/Allele**	**OR (95% CI)**	**p-value**	**AIC**
Codominant	GT *vs.* GG<br>TT *vs.* GG	0.565 (0.237-1.35)<br>0.360 (0.044-2.93)	0.199<br>0.339	173.755
Dominant	GT+TT *vs.* GG	0.531 (0.231-1.222)	0.137	171.937
Recessive	TT *vs.* GG+GT	0.431 (0.054-3.463)	0.429	173.505
Overdominant	GT *vs.* GG+TT	0.607 (0.256-1.441)	0.258	172.929
Additive	G	0.578 (0.284-1.179)	0.132	171.769
***STAT4* (rs7601754):**				
**Model**	**Genotype/Allele**	**OR (95% CI)**	**p-value**	**AIC**
Codominant	GA *vs.* AA<br>GG *vs.* AA	1.456 (0.614-3.454)<br>1.833 (0.182-18.493)	0.394<br>0.607	175.406
Dominant	GA+GG *vs.* AA	1.486 (0.646-3.42)	0.351	173.441
Recessive	GG *vs.* AA+GA	1.667 (0.168-16.554)	0.663	174.107

Overdominant	GA *vs.* AA+GG	1.427 (0.605-3.369)	0.417	173.645
Additive	A	1.419 (0.69-2.92)	0.341	173.417
***STAT4* (rs10168266):**				
**Model**	**Genotype/Allele**	**OR (95% CI)**	**p-value**	**AIC**
Codominant	CT *vs.* CC<br>TT *vs.* CC	0.412 (0.149-1.138)<br>1.010 (0.108-9.417)	0.087<br>0.993	172.871
Dominant	CT+TT *vs.* CC	0.457 (0.177-1.179)	0.105	171.369
Recessive	TT *vs.* CC+CT	1.424 (0.134-11.492)	0.849	174.247
Overdominant	CT *vs.* CC+CT	0.412 (0.15-1.133)	0.086	170.871
Additive	C	0.552 (0.237-1.286)	0.169	172.156
**Without MS**				
***STAT4* (rs10181656):**				
**Model**	**Genotype/Allele**	**OR (95% CI)**	**p-value**	**AIC**
Codominant	CG *vs.* CC<br>GG *vs.* CC	0.836 (0.403-1.732)<br>0.692 (0.143-3.36)	0.629<br>0.648	209.195
Dominant	CG+GG *vs.* CC	0.814 (0.405-1.637)	0.564	207.247
Recessive	GG *vs.* CC+CG	0.740 (0.156-3.514)	0.705	207.430
Overdominant	CG *vs.* CC+GG	0.862 (0.42-1.768)	0.686	207.417
Additive	C	0.834 (0.468-1.468)	0.538	207.195
***STAT4* (rs7574865):**				
**Model**	**Genotype/Allele**	**OR (95% CI)**	**p-value**	**AIC**
Codominant	GT *vs.* GG<br>TT *vs.* GG	0.910 (0.437-1.896)<br>0.993 (0.257-3.834)	0.801<br>0.992	209.517
Dominant	GT+TT *vs.* GG	0.924 (0.462-1.845)	0.822	207.536
Recessive	TT *vs.* GG+GT	1.028 (0.273-3.866)	0.967	207.581
Overdominant	GT *vs.* GG+TT	0.911 (0.443-1.87)	0.799	207.517
Additive	G	0.956 (0.552-1.665)	0.872	207.557
***STAT4* (rs7601754):**				
**Model**	**Genotype/Allele**	**OR (95% CI)**	**p-value**	**AIC**
Codominant	GA *vs.* AA<br>GG *vs.* AA	1.148 (0.512-2.575)<br>1.301 (0.131-12.942)	0.738<br>0.822	209.434
Dominant	GA+GG *vs.* AA	1.160 (0.532-2.532)	0.709	207.445
Recessive	GG *vs.* AA+GA	1.260 (0.128-12.434)	0.843	207.545
Overdominant	GA *vs.* AA+GG	1.140(0.51-2.549)	0.750	207.483
Additive	A	1.146(0.577-2.275)	0.698	207.434
***STAT4* (rs10168266):**				
**Model**	**Genotype/Allele**	**OR (95% CI)**	**p-value**	**AIC**
Codominant	CT *vs.* CC<br>TT *vs.* CC	1.032 (0.491-2.17)<br>1.944 (0.338-11.181)	0.934<br>0.456	209.069
Dominant	CT+TT *vs.* CC	1.101 (0.54-2.244)	0.792	207.513
Recessive	TT *vs.* CC+CT	1.925 (0.34-10.887)	0.459	207.075
Overdominant	CT *vs.* CC+CT	0.997 (0.478-2.082)	0.994	207.583
Additive	C	1.158 (0.628-2.14)	0.640	207.367

p-value – level of significance; differences were considered as statistically significant when p<0.05, OR – capability ratio; AIC – Akaike.

Table S9. Haplotype association with the predisposition to optical neuritis with MS occurrence.

Haplotype	*STAT4* rs10181656	*STAT4* rs7574865	*STAT4* rs7601754	*STAT4* rs10168266	Frequency		OR (95 % CI)	p-value
					Control	ON		
1	C	G	A	C	59.43	61.82	1.00	–
2	G	T	A	T	14.57	10.06	0.61 (0.26 - 1.43)	0.26
3	C	G	G	C	11.78	14.93	1.34 (0.63 – 2.81)	0.45
4	G	T	A	C	9.16	3.41	0.46 (0.13 - 1.59)	0.22
5	C	G	A	T	2.59	-	-	-

OR: odds ratio; CI: confidence interval; p-value: significance level (after Bonferroni correction statistically significant when p<0.0125).
